# RECESSION EXPERIENCES AND SELF-REPORTED DAILY SLEEP QUALITY IN MID-LIFE AND AGING ADULTS

**DOI:** 10.1093/geroni/igad104.1924

**Published:** 2023-12-21

**Authors:** Aarti Bhat, Sun Ah Lee, Jose Diaz, David Almeida

**Affiliations:** The Pennsylvania State University, University Park, Pennsylvania, United States; The Pennsylvania State University, University Park, Pennsylvania, United States; Penn State University, University Park, Pennsylvania, United States; The Pennsylvania State University, State College, Pennsylvania, United States

## Abstract

Adverse economic events can negatively impact various indicators of health, including sleep quality. Sleep quality issues are associated with chronic physical health conditions and poor mental health. While there has been a more established body of work on stress and global indicators of sleep, assessing sleep quality at the daily level can contribute to further understanding how stress can impact everyday life. Given that midlife and older adults are a fast-growing demographic in the United States, who have experienced economic events such as the 2007-2009 Great Recession as well as the COVID-19 pandemic, it becomes critical to study how such economic experiences affect markers of wellbeing, such as daily sleep, for aging adults. This study examines the effect of recession hardships on daily sleep quality in aging adults using the Midlife in the United States Refresher study. This study will combine the MIDUS Refresher Survey project with the MIDUS Refresher Biomarker project (n=863) to assess the contribution of adverse Recession events across financial, job, and housing experiences (86% of participants reported at least one adverse Recession experience) on quality of sleep (e.g., time to fall asleep, wakefulness during night) using the Daily Sleep Diary component of the MIDUS Refresher Biomarker project, in which participants self-reported on their sleep quality for seven days. This study will provide insight into how certain types of Recession events may differentially affect daily sleep patterns for aging adults; and provide information of within and between person differences in how Recession events impact daily sleep.

